# Modulation of ERQC and ERAD: A Broad-Spectrum Spanner in the Works of Cancer Cells?

**DOI:** 10.1155/2019/8384913

**Published:** 2019-10-01

**Authors:** Gábor Tax, Andrea Lia, Angelo Santino, Pietro Roversi

**Affiliations:** ^1^Leicester Institute of Structural and Chemical Biology, Department of Molecular and Cell Biology, University of Leicester, Henry Wellcome Building, Lancaster Road, Leicester LE1 7RH, UK; ^2^Institute of Sciences of Food Production, C.N.R. Unit of Lecce, via Monteroni, I-73100 Lecce, Italy

## Abstract

Endoplasmic reticulum glycoprotein folding quality control (ERQC) and ER-associated degradation (ERAD) preside over cellular glycoprotein secretion and maintain steady glycoproteostasis. When cells turn malignant, cancer cell plasticity is affected and supported either by point mutations, preferential isoform selection, altered expression levels, or shifts to conformational equilibria of a secreted glycoprotein. Such changes are crucial in mediating altered extracellular signalling, metabolic behavior, and adhesion properties of cancer cells. It is therefore conceivable that interference with ERQC and/or ERAD can be used to selectively damage cancers. Indeed, inhibitors of the late stages of ERAD are already in the clinic against cancers such as multiple myeloma. Here, we review recent advances in our understanding of the complex relationship between glycoproteostasis and cancer biology and discuss the potential of ERQC and ERAD modulators for the selective targeting of cancer cell plasticity.

## 1. Introduction

Plasticity, an intrinsic characteristic of healthy cells in biological contexts as varied as embryonal development [1], tissue development and repair [2], adaptation to injury [3], and wound healing [4], is also central to cancer initiation, progression, and metastasis. The proteins establishing and maintaining cancer plasticity are good anticancer drug targets in the fight against cancer initiation, progression, and therapy resistance itself [5]. Plasticity of cancer cells relies heavily on glycoproteins that traverse the secretory pathway, such as cell surface receptors and signalling molecules released in the extracellular medium [6, 7]. These secreted glycoproteins respond to and steer changes in the surroundings of a cancer cell, and contribute to tumour immunity [8], tumour growth and cancer cell division, adhesion and metastasis.

The reliance of cancer cells on secreted glycoproteins begs the question as to whether the endoplasmic reticulum glycoprotein folding quality control (ERQC) and/or endoplasmic reticulum associated degradation (ERAD) systems (together with the parallel misfolding-associated protein secretion system, MAPS [9]) could constitute potential anti-cancer targets. It is conceivable that ERQC/ERAD would make attractive targets for the treatment of cell malignancies [10], in that the fitness of the cancer cells, particularly those bearing a high secretory burden such as multiple myeloma cells [11], is critically dependent on functional integrity of the endoplasmic reticulum (ER), which in turn relies on ERQC/ERAD as ER stress-attenuating mechanisms.

The therapeutic value of pharmacological chaperones (small molecules specifically stabilising a misfolded glycoprotein as it traverses the ER) is already well established in a number of congenital glycoprotein misfolding endocrine and metabolic disorders [12], further supporting the idea that therapeutic modulation of ER glycoprotein folding and degradation systems could also be successfully applied to cancer treatment, at least in cases where ERQC-assisted glycoprotein folding and ERAD play a major role.

Importantly, while pharmacological chaperones are designed to bind individual misfolded glycoproteins, any drug targeting a specific ERQC/ERAD component would affect folding of all glycoproteins that are dependent on it for their folding/degradation. Given the unique and central role of ERQC/ERAD in the fate of hundreds of secreted glycoproteins, and remembering that plasticity of different cancers depends on different subsets of secreted glycoproteins, ERQC/ERAD modulating drugs may have the potential to represent broad-spectrum anti-cancer agents.

Of course, like any strategy aimed at inhibition/modulation of basic cell housekeeping machineries, molecules developed to interfere with ERQC/ERAD have the potential to be toxic to healthy cells as well as cancerous ones. In addition, ERQC/ERAD inhibition could lead to increased levels of prematurely secreted misfolded glycoproteins (a scenario akin to the opening of an “ER Pandora's box”).

In this review article, we explore the evidence suggesting that the ability of cancer cells to create and spread tumours around the body, to resist current therapies, and to recur post-treatment, hinges vitally on ERQC/ERAD. We review our current understanding of how ERQC/ERAD preserve ER glycoproteostasis and discuss how we may harness the molecular detail so far established on these systems in order to develop new broad-spectrum anti-cancer therapeutics.

## 2. Materials and Methods

### 2.1. Homology Modelling

The HHPred server [13] was used to align the protein sequences with the ones of orthologues of known structure and create homology models with MODELLER [14]. The transmembrane helix of *Mm*MOGS (mouse GCS1, UniProt Q80UM7, MOGS_MOUSE, residues 42–62) was homology modelled on PDB entry 1HH0, residues A20-A40. The C-terminal part of human calnexin (UniProt P27824, CALX_HUMAN, residues 461–484) was homology modelled using PDB ID 6A69, residues B223–B246. The C-terminal part of human Sep15 (UniProt O60613, SEP15_HUMAN, residues 46–134) was homology modelled using PDB ID 2A4H, residues A11-A99. The human ER UDPase (UniProt O75356, ENTP5_HUMAN, residues 22–404) was homology modelled using PDB ID 5U7W, residues A4–A412. The human UDP-Glc transporter (putatively identified as UniProt P78383, S35B1_HUMAN, residues 9–321; although a recently published paper reports ATP/ADP antiporter activity [15]) was homology modelled on the basis of PDB ID 5OGE, residues C16–C333. The ER lumenal domain of human EDEM1 (UniProt Q92611, EDEM1_HUMAN, residues 126–587) was homology modelled on the basis of PDB ID 1X9D A84-A535; its N-terminal transmembrane part, residues 1–34, was homology modelled on the basis of PDB ID 5MRW, residues E57–E90. The HRD1/HRD3 complex was modelled by docking the crystal structures (PDB IDs 5V6P and 5V7V) in the cryo-EM map for the complex (Electron Microscopy Data Bank ID EMD-8638 [16]), using Chimera [17]. All protein structure figures were made with PyMol [18].

### 2.2. ERQC/ERAD and Cancer

**Figure 1:**
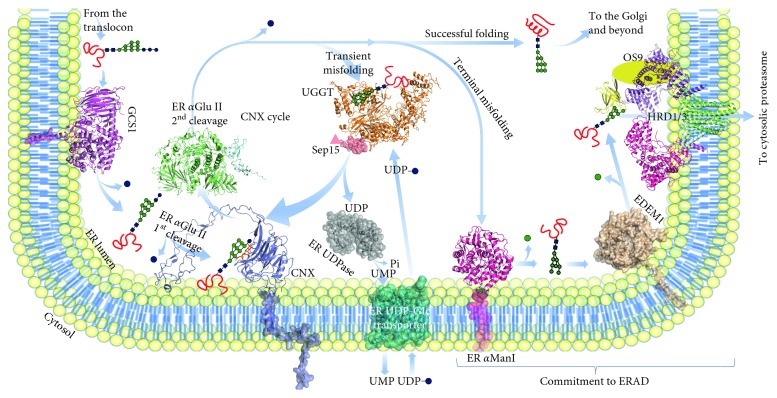
Structural view of ERQC/ERAD. The ER trajectory of the folding stages of a glycoprotein, bound to a glycan of variable composition, is followed through its interactions with ERQC/ERAD components, either ER-lumenal or ER-membrane associated. Proteins whose structures have been described in the literature for at least one eukaryotic species are in cartoon representation, while homology models of proteins of unknown structure are in semitransparent surface representation. Proteins for which no orthologue of known structure exists are represented as arbitrary 2D shapes. The red squiggly symbol represents the polypeptidic part of a glycoprotein on its way to folding (either incompletely folded, transiently misfolded, or terminally misfolded), while the red helix and two-stranded-*β*-sheet symbol represents a glycoprotein that has successfully attained its native fold. The symbols of the monosaccharides in the glycan are from [151]: blue squares, N-acetyl glucosamine (GlcNAc); green circles, mannose (Man); blue circles, glucose (Glc). The symbolic N-linked glycan on the glycoprotein is not to scale with the proteins, and the proteins themselves are not represented to exact relative scale to one another. The nascent glycoprotein in the upper-left corner has just been synthesised into the ER by a translocon-associated ribosome. After translation/translocation, the polypeptide has been glycosylated by oligosaccharyl-transferase (OST, not shown) and carries a GlcNAc_2_Man_9_Glc_3_ glycan. The nascent glycoprotein is first deglucosylated by GCS1 (purple, mouse GCS1, PDB ID 5MHF, on the left), which leaves a GlcNAc_2_Man_9_Glc_2_ glycan; a further deglucosylation step by ER *α*GluII (mouse GCS2, green and cyan, PDB ID 5F0E) produces a GlcNAc_2_Man_9_Glc_1_ glycan, through which the glycoprotein can associate with the lectin domain of either calnexin (CNX, indigo, dog calnexin, PDB ID 1JHN) or calreticulin (not shown) and the associated foldases/chaperones (not shown). A second cleavage by ER *α*GluII leaves a GlcNAc_2_Man_9_ glycan on the glycoprotein, which can bind no longer to the ER lectins and if folded is free to progress to the Golgi and beyond (upper right corner). If the glycoprotein fold is not native yet, the misfolded glycoprotein is recognised and reglucosylated by UGGT (orange, *Ct*UGGT, PDB ID 5NV4), using UDP-Glc as a source of glucose. The resulting GlcNAc_2_Man_9_Glc_1_ glycoprotein can reassociate with the CNX/CRT to profit from further attempts at chaperone/foldase-assisted folding. The UGGT partner protein Sep15 is in salmon pink (triangle: Nterm domain; surface: Cterm domain). The UDP molecule produced by UGGT-mediated reglucosylation is hydrolysed by a ER UDPase (grey) to inorganic phosphate (*P*_i_) and UMP—the latter being antiported back to the cytoplasm by an ER-membrane-resident ER UDP-Glc transporter (cyan), in exchange for a molecule of UDP-Glc. Terminally misfolded glycoproteins are first demannosylated by ER *α*ManI (magenta, PDB ID 1X9D) and then by EDEM1 (wheat), to yield a GlcNAc_2_Man_5–7_ glycan. The mannose-6-phosphate receptor homology (MRH) domain of OS-9 (yellow) binds the demannosylated glycan and recruits the terminally misfolded glycoprotein to the ERAD retrotranslocon assembled around the HRD1/HRD3 ubiquitin ligase (magenta, violet, green and cyan, yeast structures, PDB IDs 5V6P and 5V7V, on the right). A cytoplasmic proteasome then degrades the terminally misfolded ubiquitinated glycoprotein (not shown).

Glycoproteins traversing the secretory pathway of eukaryotic cells reach their cellular or extracellular destinations after folding in the ER [19]. To deal with the constant challenge of protein misfolding in the ER, eukaryotic cells have evolved the ERQC system, centred around the calnexin cycle [20]. Collectively, ERQC components (left-hand side of Figure 1) identify, retain in the ER, and aid folding of misfolded glycoproteins on the way down the secretory pathway. ERQC surveys glycoprotein folding, prevents premature glycoprotein secretion, and is integrated with the adaptive stress response [10]. ERQC proteins either reside in the ER lumen or are inserted in/associated with the ER membrane. A second ER-resident machinery called endoplasmic reticulum-associated degradation (ERAD, right-hand side of Figure 1) comprises proteins that commit terminally misfolded glycoproteins to demannosylation, retrotranslocation to the cytoplasm, and ubiquitination, ultimately targeting them to cytoplasmic proteasomes. Both ERQC and ERAD support cells in their effort to fine tune the rate of glycoprotein synthesis and entry into the ER to match the ER folding capacity (glycoprotein homeostasis or glycoproteostasis) [21].

Malignant cells are deprived of nutrients and their protein synthesis is dysregulated, so that they are especially prone to ER stress. The latter results from protein misfolding within the ER, and it has profound effects on cancer cells' proliferation and survival [22]. It is therefore not surprising that ERQC and ERAD play a key role in cancer biology. Yet, the complexity of ER glycoproteostasis, coupled with the galaxy of cancer cell phenotypes, makes it nontrivial to predict if the activity of a specific ERQC/ERAD component helps or hinders establishment and progression of a specific type of cancer. Indeed, ER quality control and degradation systems have been suggested to represent a double-edged sword that may aid progression as well as prevention of cancer cell growth in a context-dependent manner [23].

Table 1 lists a number of ERQC/ERAD components and association of their expression levels with cancer patient survival in the Human Protein Atlas (HPA) [24, 25], as evidenced by Kaplan–Meier survival plots [26] derived from cancer tissue images. Quite a few of these ERQC/ERAD components have been identified as unfavourable prognostic markers in cancer studies. We also list the frequency of somatic mutations detected in the same genes, as reported by the Catalogue of Somatic Mutations in Cancer (COSMIC), the world's largest source of manually curated somatic mutation information relating to human cancers [27]. Other useful resources are the database of therapeutic vulnerability of cancer [28], lists of oncogenes [29], and the tumour suppressor gene TUSON ranking [30] (https://bioinfo.uth.edu/TSGene/ [40, 41]) in the cancer cell metabolism genes database [31], but in the interest of simplicity, we did not compile values from these online sources in Table 1.

In the following paragraphs, we briefly review some of the published evidence of direct cancer association for a selected subset of ERQC/ERAD components, before examining the second-order involvement of ERQC/ERAD with cancer, through their regulation of folding and degradation of specific cancer-associated secreted glycoproteins.

### 2.3. ERQC and Cancer

*ER α-glucosidase I* (GCS1, in purple on the left-hand side in Figure 1) directly interacts with subunits of the ER-membrane-associated oligosaccharyl transferase (OST) [30, 31], in agreement with what was observed for the yeast orthologues [32, 33]. GCS1 acts as the porter at the ERQC one-way entrance door, removing the outer glucose (Glc) residue from the Glc_3_Man_9_GlcNAc_2_ N-linked glycan transferred by OST to a nascent glycoprotein. With this cleavage, ER Glu I generates diglucosylated glycoproteins, i.e., glycoproteins carrying Glc_2_Man_9_GlcNAc_2_ N-linked glycans. This kind of glycan in turn is necessary for the first interaction with the second major ERQC player, ER *α*Glu II: without the ER *α*Glu I-mediated Glc cleavage, glycoproteins cannot interact with ER *α*Glu II nor enter ERQC [32, 33]. A direct role for diglucosylated glycans in ERQC has also been hypothesised in conjunction with malectin, the ER lectin that binds them specifically [34]. Genetic defects in MOGS, the gene encoding GCS1, cause rare congenital disorder of glycosylation type IIb (CDG-IIb) and confer decreased susceptibility to infections due to viruses whose life cycle depends on the host cell's calnexin cycle [35]. The Human Protein Atlas (HPA) [24, 25] reports unfavourable prognoses in human renal, liver, and colorectal cancers overexpressing the MOGS gene (see Table 1).

*ER α-glucosidase II* (ER *α*Glu II, in green and cyan in Figure 1) acts as an usher, mediating both entry and exit of a glycoprotein into the cycle [36]. Entry into ERQC is conditional on ER *α*Glu II-mediated removal of the terminal Glc from the Glc_2_Man_9_GlcNAc_2_ glycan, enabling recruitment of the resulting monoglucosylated glycoprotein to the ER lectins calnexin and calreticulin, and the associated oxido-reductases, isomerases, and foldases. The same ER *α*Glu II eventually removes the remaining Glc from the Glc_1_Man_9_GlcNAc_2_ glycan, preventing further association with the ER lectins, thus freeing a glycoprotein from the refolding end of ERQC [37]. The noncatalytic ER *α*Glu II *β* subunit likely mediates association with the client glycoprotein glycan via its C-terminal mannose 6-phosphate receptor homology (MRH) domain, and it contains the ER-retrieval motif localising ER *α*Glu II to the ER [38]. Overexpression of ER *α*Glu II *β* subunit (ER *α*Glu II *β*) in different tumour tissues has been reported [40, 41]. More recently, it has been suggested that activation of ERQC through ER *α*Glu II can help tumour cells to escape from autophagy and apoptosis [42]. A study of molecular chaperones regulating the invasion phenotype of head and neck cancer (HNC) established that loss of the tumour suppression function of the ER *α*Glu II *α* subunit contributed to aggressive cancers [39].

*Calnexin* (CNX, ER-membrane inserted, in violet in Figure 1) and *calreticulin* (CRT, ER lumenal and soluble) are *ERQC lectins* with a specificity for mono-glucosylated glycans (Glc_1_Man_9_GlcNAc_2_). They recruit monoglucosylated glycoproteins to oxido-reductases, isomerases, and foldases, effectively constituting the refolding end of the calnexin cycle. In one lung cancer study, low levels of CNX contributed to poor prognosis: in a cell-culture model, targeted depletion of calnexin reduced cancer proliferation, invasion, and migration [44]. CNX expression positively correlates with metastasis of breast cancer to the brain [45]. CNX was also significantly upregulated in oral squamous cell carcinoma, and its levels correlated with poor prognosis in patients affected by this tumour [46].

**Figure 2:**
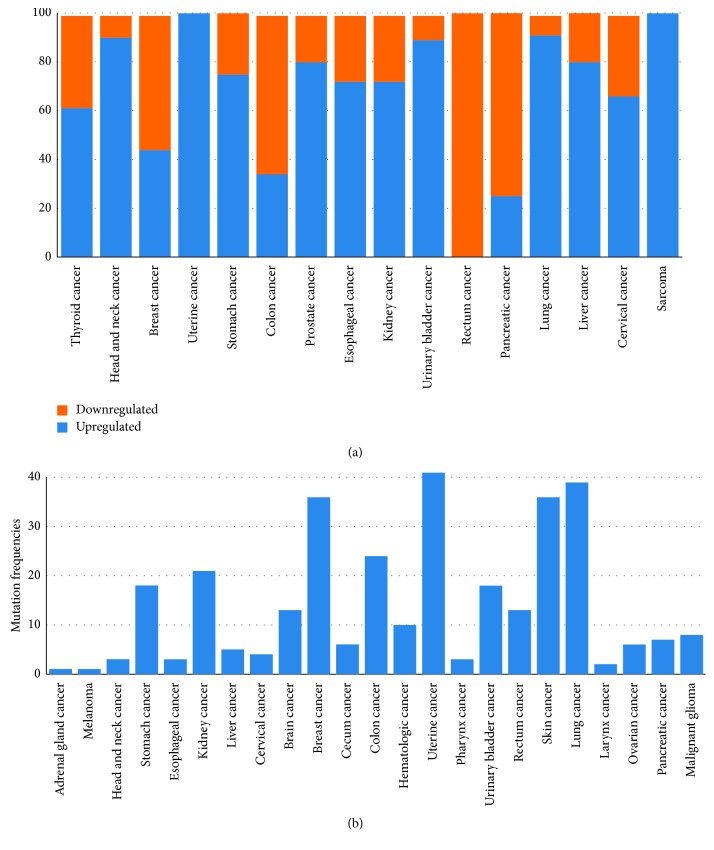
(a) Percentage of upregulation and downregulation of the UGGT1 gene in a number of cancers from RNA-seq experiments on bulk cancer tissues. (b) Mutation frequencies in various cancer types in the same gene (from the Q9NYU2 entries in BioMuta and BioXpress at the OncoMX server [152]).

*UGGT* (UDP-glucose glycoprotein glucosyltransferase) is the ERQC checkpoint, detecting misfolded glycoproteins and reglucosylating them in order to enable further rounds of association with CNX/CRT, beyond the initial one(s) afforded by the OST-transferred N-glycan(s) after the initial ER *α*Glu II cleavages [40]. In higher vertebrates, there are two UGGT isoforms, UGGT1 and UGGT2. Although UGGT2 was initially reported not to reglucosylate UGGT1 misfolded glycoprotein clients [41], this isoform is also competent in reglucosylating synthetic glycoproteins carrying high-mannose glycans [42, 43], suggesting that UGGT1 and UGGT2 evolved to act on different subsets of glycoprotein clients. The mechanism by which UGGT recognises and selectively reglucosylates misfolded glycoproteins remains unclear. The observation that UGGT bears demannosylated glycans that are the hallmark of ERAD [44, 45] is compatible with the hypothesis that UGGT may recognise misfolded glycoproteins via an intrinsically misfolded domain (“it takes one to know one”), as observed for the mouse ERAD mannosidase [46]. Despite the centrality of UGGT to eukaryotic glycoprotein secretion, only a few *bona fide* UGGT glycoprotein clients are known [47–52], and the full lists of clients of the two isoforms (“UGGT-omes”) remain to be compiled. The Human Protein Atlas (HPA) [24, 25] reports unfavourable prognoses in renal cancers and lung and liver cancers overexpressing UGGT1 or UGGT2, respectively. A majority of cancers are reported to overexpress the UGGT1 gene (see Figure 2(a)), and a few cancer types report a significant rate of mutations in the same gene (see Figure 2(b))—although without functional data, it is difficult to assess if they are likely to impair or enhance protein function. No studies have directly tested the role of UGGT in cancer plasticity.

*Sep15* (aka Selenoprotein F, Selenof) is a 15 kDa protein which in humans (but not in fruit fly, mosquito, zebrafish or rat) contains a selenocysteine residue [53]. Selenium has been implicated in cancer prevention [54], but the mechanism and possible involvement of selenoproteins in this process are not well understood. Based on the fact that abnormal glycoprotein folding and secretion were observed in conjunction with Sep15 deficiency, it has been proposed that it may have an important role in the ER maturation of N-glycosylated proteins [55], in particular M-immunoglobulins [56]. Sep15 mitigates oxidative stress and apoptosis [57]. Its C-terminal domain (residues 46–134) folds as a thioredoxin-like domain [58]; the N-terminal domain (residues 1–45), whose fold is not easily predictable from sequence, likely mediates Sep15 nanomolar association with UGGT1 [59]. Indeed, Sep15 enhances UGGT1-mediated reglucosylation of IL-8 and crambin containing mispaired disulphides [42, 43], suggesting that the Sep15 redox potential may have evolved to selectively reduce/isomerise disulphides in nonnative over native environments. A number of studies point to a role of Sep15 in cancer aetiology. The Sep15 coding gene is located in a highly mutated region of chromosome 1, and several mutations and deletions of Sep15 coding gene are involved in cancer progression and tumorigenesis [53]. The expression levels of Sep15 were investigated in various cancer models: downregulation of the protein was found in hepatocarcinomas and colorectal, gastric, and prostate cancers [53, 54, 60, 61]. On the other hand, decreased expression of Sep15 reduces proliferation and growth of liver and colon cancer cell lines, pointing to a role of Sep15 in tumour progression [60, 62–64]. Single-nucleotide polymorphisms in the Sep15 gene have been studied in conjunction with differential levels of selenocysteine insertion [65] and susceptibility to lung and breast cancer [66–68], highlighting the need for a stratified medicine approach in the development of Sep15 modulators as anticancer therapeutics.

Supply of UDP-glucose to the ER is thought to be mediated by an ER-transmembrane *UDP-Glc/UMP antiporter* (in cyan in Figure 1), in analogy with other sugar nucleotides synthesised in the cytoplasm and transported to the ER or to the Golgi by specific antiporters. Sugar nucleotide/nucleotide monophosphate antiporters (or nucleotide sugar transporters, NST for short) are a subclass of the solute carrier transporter family of molecules that have been proposed as potential targets for digestive system neoplasms [69]. Until recently, and on the basis of sequence homology to known NSTs [70], the putative gene encoding the human UDP-Glc/UMP antiporter was the solute carrier family 35 member B1 aka SLC35B1 or UGTrel1 (UniProt P78383, S35B1_HUMAN). Intriguingly, deletion of the ER-localised members of the NST family in *Schizosaccharomyces pombe* produces phenotypes similar to the deletion of the UGGT gene, but even when combined with disruption of all known NST genes whose products have an unknown location, loss of genes encoding known ER NSTs did not obliterate UDP-Glc ER entrance [71]. Last year, a study characterised SLC35B1 as an ATP/ADP antiporter [15]. These observations combined now support the hypothesis that UDP-Glc entrance into the yeast ER may not follow the classical NST antiport mechanism.

Whichever the source of ER UDP-glucose, once UGGT has transferred a Glc molecule from UDP-Glc to a misfolded glycoprotein glycan, a molecule of UDP is produced, which would inhibit UGGT [72]. As is the case for other nucleoside diphosphates produced by sugar transferases [73], an *ER-specific UDPase* (NTPD5, UniProt O75356, ENTP5_HUMAN, in grey in Figure 1) hydrolyses the ER UDP pool to UMP [72, 74]. NTPD5 may mediate some of the cancer-related phenotypes associated with AKT1 activation: NTPD5 is upregulated in cell lines and primary human tumor samples with active AKT and, together with cytidine monophosphate kinase-1 and adenylate kinase-1, is part of an ATP hydrolysis cycle that converts ATP to AMP, resulting in the cancer-associated compensatory increase in aerobic glycolysis known as the Warburg effect [75]. Many studies have correlated dysregulation of the expression of the ER UDPase with a range of cancers, explaining why the enzyme has been proposed as a potential target for anticancer therapy [76–80].

### 2.4. ERAD in Cancer

Just as the N-linked glycan is used by ERQC to add/remove the glucose whose presence/absence marks a misfolded glycoprotein for ER retention/progression to the Golgi, ERAD mannosidases remove mannose residues from the N-linked glycan, flagging a terminally misfolded glycoprotein for degradation [81]. In particular, trimming of N-glycans by ERAD mannosidases generates Man_6_GlcNAc_2_ and Man_5_GlcNAc_2_ (M6 and M5) glycans, with three main consequences [82]: (i) removal of the outer Man residues on branch A precludes reentry of the glycoprotein molecule in the calnexin cycle; (ii) the trimmed M5-6 structures bind to the lectins OS-9 and XTP-3B [83], targeting the glycoprotein to retrotranslocation by the SEL1L/HRD1 ERAD dislocon complex; and (iii) the trimmed species are selected against ER-to-Golgi transport [84]. Unlike ERQC, where the glucose residue can be put back on the N-linked glycan by UGGT and the cycle glucose-on/glucose-off repeated, no ERAD mannosyl-transferase is known, so after the first steps of ERAD-mediated demannosylation, a glycoprotein is irretrievably dispatched to degradation [85].

Correct identification of misfolded secretory glycoproteins and their degradation by ERAD are crucial for cellular health and survival. ERAD processing is not stochastic: ERAD glycan trimming is selectively accelerated on misfolded glycoprotein [82]. Without functional ERAD, misfolded glycoproteins accumulate, the ER is stressed, and the unfolded protein response (UPR) ensues. While the early UPR response tries to increase the production of molecular chaperones involved in protein folding, prolonged stress activates UPR arms steering the cell towards apoptosis.

High growth rate, impaired ATP generation, hypoxia, hypoglycemia, and specific mutations perturb cancer cells' ER homeostasis [86, 87] and may also induce UPR [88]. This in turn can lead to cell death. ERAD unwittingly (but effectively) helps cancer cells by conferring them tolerance to glycoproteotoxic stress. Indeed, survival under chronic ER stress is a feature of aggressive cancers [89], and tumour cells attempt survival by hijacking ERAD [90]. For these reasons, terminal ERAD components inhibitors have been proposed as targets to specifically impair the survival of cancer cells [22, 91]. Blocking ERAD can also trigger cellular apoptosis [92].

The ERAD components acting early in the pathway are the endoplasmic reticulum degradation-enhancing mannosidases (EDEM), committing misfolded glycoproteins to degradation. To date, no EDEM-specific inhibitors are known, and the effects of EDEM inhibition/deletion on cancer cells have not been investigated, although the generic *α*-mannosidase inhibitor kifunensine [93] increased adhesion of breast cancer cells to endothelial cells [94] and 1-deoxymannojirimycin (another broad-spectrum mannosidase inhibitor) induced cellular ER stress in a human hepatocarcinoma cell line [95].

ER mannosyl-oligosaccharide 1,2-*α*-mannosidase (*ER αMan I*, UniProt Q9UKM7, MA1B1_HUMAN, in pink in Figure 1) is an 80 kDa enzyme with a short cytoplasmic tail, a single transmembrane *α* helix localising it to the ER membrane, and an ER lumenal mannosidase domain, initially believed to selectively remove only the middle arm terminal *α*(1,2)-linked D-mannose residue from the oligomannose Man_9_GlcNAc_2_ N-linked glycan [96], for which it has an affinity of 0.4 mM [97]. More recent *in vitro* and *in cellula* data highlight that ER *α*Man I can in fact remove all four *α*(1,2)-linked D-mannose residues from the glycan, although it does have a preference for the one on arm B [82, 98–100]. A crystal structure of human ER *α*Man I in complex with a glycan has revealed the structural basis for its substrate recognition and catalysis [101]. A conserved motif within the 3′UTR of ER *α*Man I is a target of miR-125b, a microRNA frequently downregulated in numerous types of cancers, including hepatocellular carcinoma (HCC), with the expression of ERManI significantly elevated in HCC, as measured by immunohistochemistry in a liver disease spectrum tissue microarray [102].

*ER ManIA aka* mannosyl-oligosaccharide 1,2-alpha-mannosidase IA (ER ManIA, UniProt P33908, MA1A1_HUMAN)— originally annotated as resident in the Golgi—has been shown to colocalise with ER *α*Man I in quality control vesicles (QCVs) and is also implicated in targeting to ERAD [103]. In cancer, the enzyme levels showed impact on degradation of cell-surface glycoprotein involved in cell-cell adhesion and metastasis: reduced ER ManIA expression or mannosidase inhibition lead to a significantly increased adhesion of breast cancer cells to endothelial cells [94]. Conversely, ER ManIA was downregulated in metastatic hepatocellular cancer (HCC) cell lines and orthotopic xenograft tumours, in comparison with nonmetastatic HCC controls [104].

ER degradation enhancing mannosidases (*EDEMs*) target misfolded glycoproteins for degradation [105] by cleaving *α*(1,2) mannoses from the glycan and exposing Man *α*(1,6) bonded residues [106]. There are two degradation-enhancing *α*(1,2) mannosidases (MNS4 and MNS5) in *Arabidopsis thaliana* [107] and three EDEMs (EDEM1, 2 and 3) in mammals. Human EDEM1 (UniProt Q92611, EDEM1_HUMAN, in wheat brown in Figure 1) is a 74 kDa enzyme inserted in the ER membrane via an N-terminal trans-membrane helix. EDEM3 (UniProt Q9BZQ6, EDEM3_HUMAN) is also ER-localised, because it carries an ER retrieval sequence at its C-terminus. EDEM2 (UniProt Q9BV94, EDEM2_HUMAN) lacks both an ER retrieval sequence and a transmembrane region [81] so its ER localisation is less certain [108]. EDEM1 overexpression can trigger ERAD in absence of ER *α*Man I [109]. Unlike ER *α*Man I, which is active even on isolated glycans, EDEM1 is more active on misfolded human glycoprotein substrates [109–112], similarly to what was observed in yeast [113, 114]. A mouse EDEM1 N-terminal region predicted to be intrinsically disordered accelerates ERAD of tyrosinase misfolded mutants [46], suggesting that misfold can be used to recognise misfold (again, as may be the case for UGGT, one hypothesis is that “it takes one to know one”). In agreement with this model is the observation that EDEM1 may be itself subjected to ERAD [115]. The Human Protein Atlas (HPA) [24, 25] reports unfavourable prognoses in renal cancers overexpressing EDEM2 and EDEM3. A somatic variant of EDEM1 (N198I), which loses one of its five N-linked glycans, was found to confer a selective advantage to hepatocellular carcinoma cells [116].

In mouse, EDEM1 has been found in association with the ERDJ5 protein disulfide isomerase (PDI) [117, 118], and the same interaction was observed between human EDEM1 and the human *ERAD PDIs* ERDJ5 [119] and TXNDC11 [112]. To date, no structure of a EDEM : PDI complex exists. It is likely that ERAD PDIs thioredoxin-like (TRXL) domains confer them the ability to process misfolded glycoproteins in the presence of nonnative disulfide bridges: if this is the case, ERAD PDI-mediated reduction of nonphysiological disulfide bridges may help the retrograde transport of misfolded substrates through the retrotranslocation channel [117]. High levels of ERDJ5 and TXNDC11 are unfavourable prognostic markers in renal and thyroid cancers and glioma, respectively [24, 25]. Knockdown of ERDJ5 by RNA interference in neuroectodermal tumour cells increases the apoptotic response to fenretinide [120]. These data make the case for selective ERDJ5 and/or TXNDC11 modulators as novel chemotherapeutic targets. On the other hand, high levels of TXNDC11 or ERDJ5 were a favourable prognostic marker in endometrial cancer [24, 25], and overexpression of ERDJ5 sensitizes neuroblastoma cells to ER stress-induced apoptosis [121], so it is clear that inhibiting this PDI may not work against some cancers. Similar results were observed for TXNDC11, whose elevated levels of expression correlated with suppression of tumour-promoting genes [122].

Once demannosylated by EDEMs, misfolded glycoproteins in the ER lumen and membrane are recruited by the osteosarcoma 9 (OS-9) and XTP3B *ERAD lectins* [123] which direct them to the ER membrane-bound complexes assembled around E3 ubiquitin ligases [124–126]. Both OS-9 and XTP3B are localised in the ER lumen [123]. OS-9 and XTP3B specifically recognise Man *α*(1,6)-Man *α*(1,6)-Man residues on the processed C-arm of the N-linked glycan [127]. XTP3B also inhibits the degradation of nonglycosylated proteins [83]. Yet again, different studies report opposite roles of the ERAD lectins in different cancers. For example, OS-9 is highly upregulated in osteosarcoma [128] and XTP3B was found to be critical for metastatic properties of human lung cancer cell lines [129], while a long noncoding RNA suppresses pancreatic ductal adenocarcinoma (PDAC) cell invasion by increasing both mRNA and protein levels for OS-9 [130].

The ERAD E3 ubiquitin-protein ligases accept ubiquitin specifically from an ER-associated E2 ligase and transfer it to glycoprotein substrates that need degradation [131]. After ubiquitination, the p97 (*aka* VCP) ATPase helps feeding substrates to a cytosolic proteasome [132]. Most of these ERAD ubiquitin-protein ligases are poorly characterised, and only few targets for each of them, for example, for the ERAD E3 enzymes HRD1 and MARCH6 [125, 133], have been identified. HRD1 protects cells from ER stress-induced apoptosis [134], and its upregulation promotes cell migration and invasion in colon cancer [135]. Another ERAD E3 enzyme, AMFR (*aka* gp78), mediates tumour invasion and metastasis functioning as a receptor for the GPI/autocrine motility factor [136]. Modulation of components of HRD1 partners [137] has been proposed as a novel point of intervention for cancer therapies, although there is published evidence that HRD1 suppresses the growth and metastasis of breast cancer cells [138] and the decrease of HRD1 expression contributed to tamoxifen resistance in breast cancer [139] (the latter by promoting the degradation of S100A8, a divalent metal ion binding protein involved in the chemistry-drug resistance in many tumours).

### 2.5. Glycoproteins and Antiancer Strategies Focussing on ERQC/ERAD Modulation

Cancer cells survive by adjusting to ER stress, and a number of studies in the literature have pointed out that components of the ERQC/ERAD machineries may constitute anticancer therapeutic targets [120, 121]. The centrality of ERQC/ERAD to glycoproteostasis would potentially endow such compounds with broad-spectrum activity, but depending on their glycoprotein secretory burden, different cancers will vary in their sensitivity to strategies that interfere with ER stress and glycoprotein folding and degradation [10]. Importantly, as is the case for any drug that interferes with basic cellular pathways, ERQC/ERAD modulators are potentially toxic to healthy cells as well. For these reasons, the most promising use for ERQC/ERAD modulators will likely be in combination with existing chemotherapeutics. For example, inhibition of homeostatic ER stress responses enhances apoptosis induced by oxidative stress-inducing drugs acting through the ER stress pathway [120].

Any attempt to develop ERQC/ERAD modulators as anticancer therapeutics would want to aim at ER stress-mediated selective killing of malignant cells without imposing significant damage to surrounding healthy cells. To be selective in aid of anticancer therapy, any ERQC/ERAD inhibitor of this kind needs to exploit different folding requirements of specific glycoproteins in cancerous vs. healthy cells. Amongst the many glycoprotein-dependent strategies used specifically by cancer cells are the expression of tumour-specific glycoprotein isoforms (with patterns of alternative splicing of mRNAs differing between tumour and normal tissues from which they are derived [140]); tumour-specific glycoprotein conformations [141]; upregulation of membrane-embedded drug transporters mediating chemotherapic multidrug resistance [142]; and expression of surface adhesion glycoproteins involved in tissue penetration and/or metastasis in leukemic cells [143] and solid malignancies [144]. Cancer cells also rely extensively on receptor tyrosine kinases (RTK): these glycoproteins are important in squamous cell carcinomas, breast/pancreas/prostate adenocarcinomas, and malignant gliomas. Indeed, nanomolar concentration of tunicamycin, a well-known inhibitor of N-glycosylation, reduces protein levels of at least four RTKs involved in tumour cell proliferation and survival [27–29].

Glycoproteins are also central to cancer immunotherapy [145, 146]: therapeutic anticancer antibodies, their cell-surface receptors, most of their epitopes [145], and complement components [147] are all glycoproteins. Many glycoproteins also underpin cancers' lack of response to immunotherapy response [12]. Drugs altering glycoprotein secretion/degradation will alter a patient's glycosecretome, including the surface antigens targeted by immunotherapy monoclonal antibodies, Fc*γ* receptors (Fc*γ*Rs), and components of the complement system. Indeed, recent evidence has implicated polymorphisms of Fc*γ*R in the efficacy of monoclonal antibody- (mAb-) mediated therapy [148]. As the molecular basis for the opposite effects between inhibitory vs. activating Fc*γ*R resides in different intracellular phosphotyrosyl-based motifs [149], the folding/degradation requirements of different Fc*γ*Rs may differ. Unfortunately, we have only partially uncovered the roles played by ERQC/ERAD during anticancer mAb therapy and, in particular, the folding and stability of cancer-specific surface glycoprotein epitopes, Fc*γ*Rs and complement components [8]: the hypothesis that drugs that selectively impair glycoprotein folding and degradation may aid cancer immunotherapy remains to be tested.

## 3. Conclusions

A large number of published studies have highlighted the dependency of a number of cancers on specific ERQC/ERAD components, but the lack of specific inhibitors of the components in both pathways has hampered proper characterisation of the roles played by ERQC/ERAD in cancer biology. Even if such specific inhibitors were available, in order to make a convincing case for ERQC/ERAD as valid anticancer targets, several aspects of ERQC/ERAD biology in healthy and cancer cells need to be better elucidated.

For example, only a few *bona fide* glycoprotein clients of ERQC/ERAD are known [150] and none of the glycoproteins with proven roles in cancer biology have been tested for their dependency on ERQC/ERAD. As the checkpoint enzymes of both machineries are likely to be critical ones, useful first pieces of knowledge towards gauging the potential of ERQC/ERAD as anticancer targets would be the lists of substrates of UGGTs and EDEMs (which collectively we call “UGGT-omes”/“EDEM-omes”), in healthy cells and in their corresponding cancer counterparts.

Other important open questions involve the degrees of redundancy and interplay between ERAD and ERQC checkpoints (again, UGGTs and EDEMs) in deciding the fate of a specific misfolded glycoprotein. Whether there is a general mechanism by which the dilemma ER retention vs. secretion is solved or whether different individual glycoproteins are taken care by the ERQC and/or the ERAD branch to different extents during their lifetime in the ER still remains to be elucidated. The extents to which specific cancers tip the EQRC/ERAD balance for glycoproteins that are crucial to their survival will be of course one of the next big questions to answer, ultimately helping in each case to make choices between ERQC vs. ERAD modulation for the most effective anticancer prescription of this kind.

Last but not least, when it comes to toxicity, although evidence of ER-retention and/or ER associated degradation exists for a few cancer-associated glycoproteins, we do not know which EQRC/ERAD clients would risk premature and unwanted secretion in healthy cells (a scenario we dubbed the “ER Pandora's box”) upon administration of an ERQC/ERAD modulator. Thus, the relative toxicity of such drugs to healthy vs. cancer cells is difficult to predict. Targeting ERQC/ERAD may well prove a broad-spectrum spanner in the plasticity works of cancer cells, but—as it often happens with cancer biology—winning this battle will require a better understanding of the roles that these machineries play in cells at various stages of the cell cycle (in healthy cells as well as in cancer tissues). Only then may ERQC/ERAD inhibitors reach the clinic, adding to the expanding arsenal of anticancer therapeutics.

## Figures and Tables

**Table 1:** ERQC/ERAD components and association of their expression levels with cancer patient survival in the Human Protein Atlas (HPA) [24, 25].

Protein/gene and UniProt entry/name	Prognosis upon overexpression (Human Protein Atlas, *p* < 0.001)	Frequency of somatic mutations in cancer (COSMIC)
GCS1/MOGS, Q13724/MOGS_HUMAN	Unfavourable prognosis in renal, liver, and colorectal cancers	191/47211 (0.4%)
ER *α*Glu II *α* subunit/GANAB, Q14697/GANAB_HUMAN	Unfavourable prognosis in liver and urothelial cancers	254/47211 (0.5%)
ER *α*Glu II *β* subunit/PRKCSH, P14314/GLU2B_HUMAN	Unfavourable prognosis in renal cancer	191/47211 (0.4%)
UGGT1/UGGT1, Q9NYU2/UGGG1_HUMAN	Unfavourable prognosis in renal cancer	333/47297 (0.7%)
UGGT2/UGGT2, Q9NYU1/UGGG2_HUMAN	Unfavourable prognosis in lung and liver cancers	406/47212 (0.8%)
Sep15/Sep15, O60613/SEP15_HUMAN	Unfavourable prognosis in liver, head, and neck cancers but favourable prognosis in colorectal cancer	17/47187 (0.04%))
Calnexin/CANX, P27824/CALX_HUMAN	Favourable prognosis in colorectal cancer but unfavourable in thyroid cancer	151/47211 (0.3%)
Calreticulin/CALR, P27797/CALR_HUMAN	Favourable prognosis in ovarian cancer but unfavourable in renal cancer	4344/81169 (5.3%)
ER UDPase, O75356, ENTP5_HUMAN	Favourable prognosis in renal cancer	110/47209 (0.2%)
ER *α*Man I, Q9UKM7, MA1B1_HUMAN	Unfavourable prognosis in liver cancer	178/47354 (0.4%)
EDEM1, Q92611, EDEM1_HUMAN	N/A	141/47255 (0.3%)
EDEM2, Q9BV94 EDEM2_HUMAN	Unfavourable prognosis in renal cancer	162/35626 (0.4%)
EDEM3, Q9BZQ6 EDEM3_HUMAN	Unfavourable prognosis in renal cancer	231/35629 (0.6%)
ERDJ5, Q8IXB1, DJC10_HUMAN	Favourable prognosis in endometrial cancer but unfavourable in renal and thyroid cancers	205/47347 (0.4%)
HRD1, Q86TM6, SYVN1_HUMAN	Favourable prognosis in head and neck cancer	143/47298 (0.3%)
OS-9/, Q13438/OS9_HUMAN	N/A	188/47797 (0.4%)

The HPA correlation (*p* < 0.001) between high levels of expression of the protein with the survival rates of cancer patients is reported, together with the frequency of somatic mutations detected in the same genes, as per the Catalogue of Somatic Mutations in Cancer (COSMIC) [27]. For comparison, the tumour suppressors TP53 and CDKN2A have mutation frequencies of 25% (40416/160297) and 6% (6067/100370), respectively.
